# Challenges and opportunities from the COVID-19 pandemic in medical education: a qualitative study

**DOI:** 10.1186/s12909-021-02682-z

**Published:** 2021-04-29

**Authors:** Ali Asghar Hayat, Mohmmad Hasan Keshavarzi, Soolmaz Zare, Leila Bazrafcan, Rita Rezaee, Seyed Aliakbar Faghihi, Mitra Amini, Javad Kojuri

**Affiliations:** Clinical Education Research Center, Shiraz University of Medical Sciences, Shiraz, Iran

**Keywords:** COVID-19 pandemic, Opportunities, Challenges, Medical education

## Abstract

**Introduction:**

Since the onset of the COVID-19 pandemic, many higher education and health centers have faced challenges. Educational leaders have tried to manage the new situation, but the human infrastructure was not ready for such an event. This study aims to explain the challenges and opportunities of the COVID-19 pandemic for medical education.

**Method:**

This qualitative study used conventional content analysis to collect data from face-to-face and semi-structured interviews. The interviews continued until data saturation was reached. The participants were 12 students and 14 faculty members at Shiraz University of Medical Sciences. To ensure data rigor, we used member checks, peer checks and an external observer.

**Results:**

Three main categories and 15 subcategories were extracted. The findings showed that four subcategories, e.g. perception on feasibility of e-learning, standardizing of e-learning, dedicated teaching, and networking and interdisciplinary collaborations, affected the development of medical e-learning. The main opportunities from the COVID-19 pandemic for medical education were classified into five subcategories: attitudes to e-learning and adaptability, preventing students’ separation from the educational environment, documentation and monitoring education, take control of own learning, and increasing perceived usefulness. The main challenges were divided into four subcategories, e.g. noncompliance with virtual classroom etiquette, inadequate interactions, time limitations, and infrastructure defects and problems. Finally, participants believed that methods of evaluation in e-learning were more suitable for diagnosis and formative evaluations. Generally, two subcategories were extracted, e.g. formative and summative.

**Conclusion:**

Medical schools have necessarily moved towards e-learning to compensate for the interruption in classroom education, such that traditional classes have been replaced with e-learning. These rapid, extensive changes in teaching and learning approaches have consequences for medical schools.

## Introduction

In late December 2019, a new coronavirus appeared in Wuhan, China [1], and spread rapidly to other parts of the world [2]. The first cases of SARS-CoV-2 infection in Iran were reported on February 19, 2020, and the number of patients had increased to 429,193 by September 22, 2020 [3]. On March 11, 2020, the World Health Organization officially declared the outbreak to be pandemic [4]. On September 22, 2020, the number of cases of infection worldwide exceeded 31 million, and more than 215 countries were affected by the virus [5]. The virus affected the daily lives of many people around the world and had negative effects on all aspects of human life –effects that were unprecedented for most people [4].

One of the areas most affected by the virus is education [6, 7], which has been halted or slowed dramatically [8] by restrictive laws and the establishment of social distancing. Educational institutions have been closed in 182 countries [6], and conventional university education has been hindered [4, 7]. In addition, more than 90% of the world’s student population has been affected by the virus [6], and the pressure on higher education systems to change their approach to distance learning (e-learning) has been maximized [2]. In response to this threat, all educational systems and professionals are trying to act appropriately by finding effective solutions to minimize the adverse effects of the pandemic on the field of education [9, 10].

This challenge is compounded in medical school, as it has not only led to an increase in demands for clinical and administrative assistance from medical schools [11] but has also put additional pressure on these institutions to adopt appropriate teaching strategies for medical students [12]. In other words, in this situation, in addition to their crucial role in combating this epidemic, medical schools and health professionals must ensure that their educational programs remain appropriate and effective [13]. Moreover, they need to maintain high-quality education for students at all levels. For this reason, universities and medical schools have suspended face-to-face, classroom-based teaching and regular tutorials in order to reduce the risk of infection [4], and have forced professors and students to use online and virtual education until the epidemic ceases. In other words, traditional education patterns have never been challenged in this way [11, 14].

In Iran, with the outbreak of the disease, all medical universities suspended face-to-face classes, and education has continued in virtual environments. Thus, the classes in this period were held both online and offline via pre-recorded lectures. In online classes, due to the speed limit of the Internet, usually, only the voices of the lecturer and the student were exchanged. In the offline format, instructors usually record narrations on their PowerPoint slides and upload them on the LMS for student access.

By May 2020, more than 5393 courses, 6971 students per course, and 12,231 professors per course have been registered or invited to register in the Navid system [15]. This system has several capabilities, including the possibility of holding simultaneous online or offline classes, uploading contents, and so on.

The COVID-19 pandemic is not the first experience to affect education, especially medical education; the SARS epidemic of 2003 impacted education, albeit to a less drastic extent [16, 17]. However, the effects of the COVID-19 pandemic will be much broader and long-lasting. Therefore, studying the challenges and opportunities created by the current pandemic in medical education can help us adapt more effectively to the new conditions, and ensure the continuation of education. It will also help prepare us to minimize disruptions in medical education in the event of an emergency. This poses a special opportunity for medical education faculties to examine the impact of the crisis on medical teaching and training, and to ensure quality medical education even during an epidemic [18]. Thus, in addition to the need to identify challenges for immediate elimination to minimize damage, such crises also provide an opportunity for faculties to use new technologies in medical education. Therefore, the present study examines the challenges and opportunities created by the COVID-19 pandemic in medical education.

## Methods

### Setting

The present study was conducted at Shiraz University of Medical Sciences, one of the most important medical education centers in southern Iran. This public university, located in the city of Shiraz, was founded in 1946 and consists of various faculties such as the school of health, medicine, nutrition sciences, new medical sciences and technologies, virtual education, nursing and midwifery, paramedical, rehabilitation, pharmaceutics, dentistry, management and information. The university currently comprises 17 faculties with more than 10,000 students, 200 majors, 782 faculty members, 54 research centers, 13 educational hospitals, 49 medical hospitals, and 32 health care networks, and provides health care services to more than 4 million people. In addition to educational and research activities, the university provides services to patients and the needy by providing complex therapeutic activities such as liver, heart and kidney transplantation, along with other advanced therapies, and serves as one of the largest and most prestigious universities in Iran.

### Participants

This qualitative study used a content analysis approach. Participants in this study were selected purposefully. The inclusion criteria were being a faculty member in basic and clinical sciences or being a medical school student, experience dealing with cyberspace technology and tools, and willingness to be interviewed. A total of 26 interviews were conducted (12 students and 14 faculty members) with 14 men and 12 women who ranged in age from 21 to 57 years.

Most of the interviewed students were studying basic sciences (7 participants) and some were externship (3 participants) and internships (2 participants), including eight men and four women with the age range of 21–25 year.

Codes, subcategories, and categories were explored through an inductive process. Categories were formulated to reflect the participants’ experiences. The researchers themselves are specialized in medical education and virtual education.

### Data collection and analysis

Data were collected during 6 months in 2020. Initially, semi-structured interviews along with field notes were used as data collection strategies. Purposeful sampling continued until saturation, meaning no further data were obtained on the topics of interest. Each interview lasted an average of 35 min. All interviews were recorded with a voice recorder and were transcribed (verbatim) immediately after they took place. The interview guide included a shortlist of general questions; for example, “How would you describe your experience in virtual education and content production?”, and “What problems did you face during the work?”. These questions were chosen based on people’s experiences working with cyberspace. For example, interviewees were asked to provide more examples or to clarify their reasons for describing the topic in question. To acquire more data, the interview continued with probing questions such as, “How do you think these challenges occur?”, “Why shouldn’t this be the case?”, and “What do you mean by that?”

The lead researcher listened to the interviews several times to obtain a general idea of the data. Ambiguities were resolved by checking the transcripts with the participants immediately after the interview or on the spot during the interview. The units of meaning that were directly related to the research question were then highlighted and selected. Subsequent analysis was performed according to Graneheim and Lundman [19]. As in other qualitative research, data collection and analysis were performed simultaneously during the study period. To identify similarities and differences, read and compared the initial codes several times. Each interview was carried out by the first author in Persian, and was literally transcribed. As a result, categories and subcategories were defined around abstract concepts related to challenges and opportunities in medical education.

### Rigor

To assess data rigor, the criteria of credibility, dependability, confirmability, and transferability were used. Data validity was investigated with a triangulation strategy. A specialized medical training team was used to investigate the findings. In addition to semi-structured interviews, note-taking during interviews and long-term data engagement were used to validate the data. The initial findings of the study, along with the codes and initial categories, were presented to a subsample of participants to solicit their opinions and feedback (member checking). Some parts of the data were peer-reviewed by colleagues who were not involved in the study (peer checking). To determine the dependability of the findings, the views of an external observer were sought. The external observer was a researcher who was familiar with e-learning and qualitative research methodology, but who was not a member of the research team. This external review confirmed the consistency of the results. To determine the confirmability of the findings, all activities were recorded and a report of the research process was prepared. In order to determine transferability, the results were shared with two non-researcher faculty members whose situations and experiences were comparable to those of the participants, and the results were again confirmed.

### Ethical approval

Permission to carry out the present study was obtained from the Ethics Committee of Shiraz University of Medical Sciences (IR.Sums.REC.2020.377). When participants were provided with information about the objectives of the research and asked to give their permission to make audio recordings of the interviews, they were assured that the information gathered would remain confidential. They were also informed that they could leave the study whenever they wished. Informed consent forms were also obtained from all participants and they were assured that leaving the study would not have any negative consequences for them.

## Results

The experiences and perceptions of faculty members, students, and other people involved in e-learning during the COVID-19 pandemic were extracted and analyzed. Based on the results of the analysis, 16 subcategories were extracted within three main categories: factors effective in the development of medical e-learning, advantages and disadvantages, and evaluation of e-learning (Table 1).

**Table 1:** Code, subcategories and categories

Categories	Subcategories	Code
Factors effective in the development of medical e-learning	Perception on feasibility of e-learning	Content type, complexity, theoretical or practical focus, feasibility of virtualization
	Standardizing of e-learning	Determining standards, online or offline presentation, multimedia use, determining the quality of delivery, determining expected minimums, avoidance of individual preferences
	Dedicated teaching	Teams for technical support and troubleshooting, user-friendly software, internship, preparing and supporting people, providing development opportunities, organizing educational resources, providing feedback.
	Networking and interdisciplinary collaborations	Group participation, international interaction, intersectional cooperation, presence of scholars in programs, participation of diverse people
Advantages and disadvantages	Attitudes to e-learning and adaptability	Decreased individual resistance, improved attitude, engagement in e-learning, developing e-learning, more flexibility, attitude change, getting used to e-learning, improving digital media skills and knowledge, familiarity with e-learning methods, learning different software, different proficiency levels
	Preventing students’ separation from the educational environment	Similarity of e-learning to face-to-face classes, increasing educational engagement, not being separated from the learning process, keeping people in the educational environment, self-directed learning
	Documentation and monitoring of education	Recording educational activities, more monitoring of educational activities, saving learning and teaching activities, possibility of lesson retrieval and review
	Take control of own learning	Ability to reviewing content, coordinated learning according to individuals’ time and pace, reflection on content and procedures, ability to pause and think about the subject
	Increasing perceived usefulness	Perceiving the usefulness of e-learning, positive attitude towards e-learning, believing in the effectiveness of virtual learning
	Noncompliance with virtual classroom etiquette	Doubts about behaviors in virtual space, unfamiliarity with virtual learning, lack of students’ commitment to presence, lack of punctuality, inappropriate clothing, lack of professional behavior
	Inadequate interactions	Interactional clinical teaching, inadequate opportunities for participation, lack of face-to-face interaction, one-sided discussions
	Time limitations	Time-consuming content preparation, time shortage, spending more time on teaching, time allocation for learning software
	Infrastructure defects and problems	Low internet speed, problems with uploading contents, problems with downloading information, weak support, web quality, inadequate computer hardware, software problems
Evaluation of e-learning	Formative evaluation Summative evaluation	Diagnostic evaluations, mechanism of individual assessment, holding open-book tests, constant, continuous and regular student assessment, developing high-taxonomy-level questions

### Factors effective in the development of medical e-learning

According to participants’ experiences and perceptions, the underlying factors detailed below interfered with useful and effective e-learning presentation. They were classified into four subcategories, including perception on feasibility of e-learning, standardizing of e-learning, dedicated teaching, and networking and interdisciplinary collaborations.

#### Perception on feasibility of e-learning

Based on participants’ experiences, given the variety of and differences in courses offered in the medical field, the volume of virtualized content of a given course should be determined first, before content virtualization. Some interviewees believed that certain medical courses, especially clinical ones and bedside medical teaching, cannot be delivered in a virtual medium. This view was most prominent for teaching the procedures.

It should be noted that the quotations of students and teachers are marked with the letters S and T, respectively.

One participant said (T3):

*“The problem is that not all courses can be taught virtually, for example, history taking, physical exam, and bedside teaching cannot be done virtually*.”

Another interviewee said (T6):

*“We have to investigate to what extent medical education can be delivered virtually. It might be possible to virtualize 100% of the content in the law field, but in medicine, this is not possible.”*

According to the experiences of participants, factors such as whether a course is theoretical or practical and clinical or nonclinical affect content virtualization and the mode of delivery. One of the participants described this topic as follows (T9):

*“The practical actions should be in-person for sure and cannot be conducted virtually, but e-learning is better in terms of theoretical concepts and clinical reasoning, and so on. It is even much better in the case of differential diagnosis or discussion of complicated clinical cases, but practical clinical teaching should be in-person.”*

The complexity of the topic was also highlighted. One of the participants said (T1):

*“I think we have a problem with the embryology course because it has a 3-dimensional mode and needs more explanation. I can’t do it virtually.”*

#### Standardizing of e-learning

One essential factor, according to participants’, was to determine the standard of teaching before presenting it. Determining the minimum standards can not only uphold the quality of teaching but also prevent nonscientific and poor delivery of teaching.

One of the participants said (T4):

*“E-learning in clinical teaching needs quality control for sure. Some standards are needed to assure quality.”*

To create e-learning content we need guidelines and a set of common rules. The type of content should be considered (T2):

“*It is better to determine the standard for how the courses are offered; otherwise, it will lead to poor quality, individual taste and students’ dissatisfaction*.”

Another participant said (S3):

*“Another problem, in this case, is the absence of a similar procedure, and the professors teach in a way that they feel comfortable with. There should be a specific platform that forces everyone to teach the content accordingly, so that one professor cannot send a spoken recording and the other slides.”*

#### Dedicated teaching

Participants believed that teaching and empowerment played an important role in the development of e-learning in medical education. According to them, faculty members should be trained to be able to attain a minimum level of skill in e-learning. There should be teams for technical support and troubleshooting for students and professors while considering and respecting their scientific knowledge.

In this connection, one participant said:

*“Because virtual education is new for most professors and students, it is better to have experts and support teams to identify and define their problems and give them respectful feedback (T12).”*

The other interviewee said:

*“Another effective thing in virtual teaching is individual training and development. If both sides receive some training in virtual systems, I think the rate of participation increases because awareness always increases things (S6).”*

#### Networking and interdisciplinary collaborations

According to the participants, if e-learning can be designed internationally or through multicenter efforts, and can provide the possibility for scholars to take part in discussions virtually, professors and students will be more satisfied.

One of the interviewees said (S2):

*“Previously, there were four professors and 20 residents in a grand round, but now we have rounds with 80 people, two in America, two in Europe, and one in Tehran. It elevates the level. When an expert, with published articles in this field, participates in the discussion, everybody participates more eagerly. Before it was not the case that professors participated in all sessions. Now, although it is not their field, some of them like to participate. Because the professors can express their idea, the percentage of professors and students who are attracted increases in educational sessions.”*

### Advantages and disadvantages

According to the participants, the sudden, compulsory implementation of e-learning in medical education after universities were closed has been followed by benefits and challenges for all stakeholders. We classified these into 9 subcategories: five for advantages and four for disadvantages.

#### Attitudes to e-learning and adaptability

Participants’ experiences reflected the view that there was resistance to accepting e-learning and using it in the university because it was slow and faced difficulties initially as a consequence of compulsory closure of universities, the novelty of online teaching, and the unfamiliarity of some faculty members with e-learning.

Also, some believed that the level of capability and virtual literacy differed among faculty members, such that the younger ones were more successful than older professors in using different types of software and producing the content. Gradually, as the knowledge and skills of those involved in providing and using existing infrastructure increased and the percentage participation of teachers and students also grew, there have been some developments in this field.

One of the interviewees explained this as follows (T10):

*“Anyway, corona led colleagues to become familiar with this approach. We learned some software. I also was forced to experience virtual software. Well, the university had this policy before, but corona forced us to do it.”*

Another advantage that e-learning created for faculty and students was holding postgraduate dissertation defense sessions online. One of them said (T5):

*“Of course, this pandemic made us look at e-learning more specifically. Now, we have the possibility of online defense sessions and conference proposals. On the day of the defense session, people enter it through the website. Panel members log in different places, and the actual defense session begins. This was considered impossible before. Now, we handle it well. I think it will continue even after corona has ended.”*

Getting used to such training has led to the acceptance of e-learning rather than in-person attendance by students and faculty. One of them said (S8):

*“Almost one week after beginning university and the academic semester, it was closed inevitably. In-person instruction was not possible anymore. After several weeks of interruption, e-learning gradually began, and the content was uploaded. It was very difficult in those two weeks. Self-study and self-learning were difficult. Yet after that, the lessons were uploaded steadily. Well, we got used to it.”*

One of them said (T13):

*“The professors, especially more experienced ones, are not familiar with the technology. For example, if they are asked to install software on their computer, they will ask for help. Therefore, high-tech professors are more successful. I have to get their videos and offer them by myself.”*

Of course, based on the experiences of most participants, most faculty members were able to succeed in this area and improve their knowledge and skills. Another participant explained the experience as follows (T10):

*“I can divide it into a pre-corona and post-corona period. The electronic literacy of the professors may have been about 10% before corona, but after that, they were forced to learn. Now, most colleagues are familiar with one method of preparing electronic content.”*

#### Preventing the separation of students from the educational environment

Due to the widespread closure of educational centers at the beginning of the pandemic, there was a concern among education stakeholders that this issue would cause students to be separated and away from the educational process and environment. Research participants believe that, gradually, with the use of e-learning capacities and a variety of virtual learning and simulation tools, students and faculty became more involved and enthusiastic in the learning process. This alleviated the initial concerns about the separation of students and faculty from the teaching and learning process and educational environment.

One of the participants said (T8):

*“E-learning should be encouraged so that the student does not feel separated. If the students are told that we will have an exam on these 20 content areas at the end of the term, students give up learning and will study lessons at the end of the semester.”*

In this regard, another participant asserted:

*“At the beginning of the university closure and the use of e-learning, students did not participate in the discussions, the instructors made long speeches without involving others. Later, by using the properties of the software and mastering them more, students participated more actively in virtual classes. For example, in some lessons, we had web groups where students had hot scientific discussions with the professor*s.”

#### Documentation and monitoring education

E-learning has provided an opportunity to promote instruction quality, thanks to increased documentation. One of the interviewees said (T5):

*“E-learning is a good opportunity. Education has to be documented now. The professor delivers it virtually so that others can see it. It will remain. If you upload a voice recording, PowerPoint file, and other things, it will be recorded. It wasn’t so before.”*

#### Take control of own learning

Participants’ experiences indicated that given the storage of e-learning and content by faculty, they could reflect on it more while reviewing it. One of the participants said (S9):

*“Since e-learning has launched, we can have the professors’ words and lessons recorded, unlike in the past. This has allowed me to play it back and review it so I can analyze and interpret it better. I think it brought me deeper learning.”*

#### Increasing perceived usefulness

Some of the participants noted that before starting e-learning sessions, they had doubts about the effectiveness and usefulness of this approach. Yet gradually, their attitude toward virtual approaches changed. For example, one of the interviewees said (T1):

*“When I was asked to hold classes virtually, I resisted a lot at first because I believed that e-learning could not be a good alternative to traditional classes. After some sessions and using different software as well as observing students’ activities, I figured out that I can obtain good outcomes with e-learning as well. I prefer to use such capabilities even in normal situations now due to their specific advantages.”*

As explained above, the participants also mentioned the disadvantages of e-learning, examples of which are discussed in detail below.

#### Inadequate interactions

One of the main concerns of participants was the creation of effective interactions for maximum learning. One of them said (S12):

*“The professors often upload offline sessions which are one-sided, and we cannot actively participate in practical terms. The professor puts a voice-over on slides and sends them. This cannot be like classes where you can raise your problems and ask questions. Therefore, students’ participation and interaction are not seen in my point of view, and that is a big problem.”*

#### Noncompliance with virtual classroom etiquette

According to most participants especially teachers, this drawback was related to the lack of students’ commitment to attendance and professional behavior in virtual classes in comparison to in-person classes. One of the participants said (T4):

*“In any case, the student feels more committed to in-person classes. Regular sleeping time, waking up on time, wearing appropriate clothes, and being on time at the university are important to students. Even if they do not interact in classes and learn few things, they are mentally committed to be there. Yet, we do not have any of this in e-learning. All educational and non-educational features of in-person classes support students in all aspects, but this is not so in virtual classes.”*

#### Time limitations

According to participants’ experiences, providing daily, updated content needed more time than in-person classes. Moreover, they believed that they had to spend more time on students’ learning via online classes. One of them said (T7):

*“We have time problems as well because the same course in online classes takes one and a half times as much time to prepare than in-person classes. For example, I teach electrocardiograms in one class but yesterday I had to explain it for one and a half hours because the students could not understand, and I had to repeat it. Therefore, it takes us more time.”*

One of the students stated:

"*In face-to-face classes, when the instructor was teaching a problem, it was better possible to ask our questions to the teacher or other students and to interact to fully understand the subject. I mean, these questions and answers led to better learning, but this is not very possible for us in virtual classrooms."*

#### Infrastructural deficits and problems

Infrastructural deficits and problems were major challenges to e-learning, and this was frequently mentioned by participants. They reported unpleasant experiences with slow internet speeds, uploading materials and files on e-learning systems, and downloading them. For example, one of the interviewees said (S5):

*“The slow speed of the internet for e-learning was one of the problems. The slow speed of downloading educational films and lessons has made everything difficult because some of the files are so large that it takes a long time for them to download. Some professors use social media messaging apps (e.g. Telegram) to send their educational files, although they are now blocked by the government. We were supposed to receive free internet, which has not happened yet.”*

Another interviewee said (S2):

*“If we have concerns over the quality of education, then attention should be given to the infrastructure. Take the internet speed; we cannot download an educational film. One of the professors uploaded a film which nobody could download.”*

#### Evaluation of e-learning in medical education

In this part, the meaning of evaluation is student evaluation and we in Iran use this term for this purpose.

The participants believed that methods of evaluation in e-learning were more suitable for diagnosis and formative evaluations, which are continuous processes.

Virtual evaluation is a new phenomenon in the university. Before the pandemic, virtual evaluation was not common at the university, nor was the evaluation infrastructure and processes provided. Therefore, virtual evaluation faced major challenges, so that the validity and reliability of its results were sometimes controversial. Therefore, the participants believed that in order to evaluate students, professors should evaluate them during the semester and, if necessary, organize face-to-face tests at the end of the semester, while following social distancing, or to use virtual testing tools with high taxonomy to reduce cheating.

One of them said (T8):

*“Some systems can be designed. The exams should be held in-person even with social distancing.”*

Unsurprisingly, some interviewees considered using open-text tests as a problem. One of them said (T14):

*“Designing such questions is very difficult; that is, you have to design high-taxonomy-level questions that students cannot answer even if they consult an open book. There are not many professors who can design these questions.”*

Some interviewees also recommended assessment tool in education, although involves some challenges.

It is necessary to evaluate students continuously during the teaching process. It is better for the faculty members to evaluate the students in each session to decide a major part of each student’s grade. Due to the coronavirus outbreak, it is no longer possible to take exams and provide assessments as before. One participant said (T11):

*“For summative tests, we should locate students in the hallways with social distancing and hold live exams on camera. There is another problem here: cheating on the exam. The student gets someone else to attend the exam. The story is very complicated.”*

## Discussion and conclusion

This study investigated the experiences of faculty members and students in medical e-learning during the sudden COVID-19 pandemic at Shiraz University of Medical Sciences. The results disclosed three main categories of responses: factors that were effective in aiding the development of medical e-learning, opportunities and challenges, and the evaluation of e-learning. The factors that affect the development and implementation of e-learning were further classified into four subcategories as discussed below.

The first effective factor was the perception on feasibility of e-learning, which reflects the extent to which courses can be delivered virtually. Medical school courses cover a large variety of different subjects. Before course contents can be virtualized, it should first be determined how much content can be virtualized successfully. Based on participants’ experiences, they identified essential considerations as the theoretical or practical and clinical or non-clinical nature of a given course, the learner’s scientific level, the complexity of the concept, the availability of required software, and the teaching method. Particularly, in practical courses, reliance on e-learning alone was viewed as inadequate to meet students’ learning needs. Therefore, a similar pattern and specific percentage of e-learning cannot be used for all courses.

The second effective factor was the standardizing of e-learning. Among the elements within this factor were possibly biased and variable ideas of faculty members based on their personal preferences, ability level, and virtual literacy, commitment, and the amount of allocated time. Students noted that these elements led to different, variable ranges of e-learning quality. Their responses also showed the importance of determining standards and teaching them to faculty members. Therefore, determining minimum standards on the basis of learners’ expected performance as well as the type of content in a given course might not only guarantee the quality of teaching, but might also prevent the influence of teachers’ individual preferences. Another effective element identified as playing a significant role in students’ learning, instruction quality, and satisfaction was content format, e.g. online and offline content, and using multimedia. In this connection, several studies have shown that relationship quality, course quality, service quality, and e-learning system quality were related to users’ and students’ satisfaction with e-learning [20–22]. Naturally, users’ satisfaction is directly and significantly affected their tendency to use e-learning [23–25] and acceptance of e-learning [24, 26].

The third effective factor was dedicated teaching. Based on the results of this study, appropriate preparation and empowerment of actors can play a pivotal role in medical e-learning. Successful, timely implementation of medical e-learning during the COVID-19 pandemic could be facilitated, according to participants, by ensuring a minimum level of required abilities in faculty members, preferring simple software for online instruction, and making technical support and troubleshooting teams available to teachers, while respecting their scientific knowledge of the course contents. Moreover, given faculty members’ unfamiliarity with the complexity of different software options and the pressures of the pandemic, using only a single software and teachers’ lack of skill with it may permanently discourage them from accepting e-learning. Therefore, an important consideration is that faculty members need practice, preparation, and support to teach effectively with e-learning technology [27, 28].

The fourth effective factor centered on networking and interdisciplinary collaborations. The e-learning experiences described by some faculty members and students indicated that greater participation and interaction were associated with more satisfaction and effectiveness. The results of this study thus suggest that this factor may have a significant impact on the quality of a given course. Thus, designing virtual education contents internationally or through multicenter collaboration appeared to lead to greater satisfaction for both faculty and students. Naturally, the presence and involvement of different people may increase their interaction and improve the quality of courses. Participation by more contributors may also increase individuals’ motivation to take part in these courses. Researchers also noted the important role of interaction and participation in the e-learning environment.

The second main category extracted from qualitative analysis comprised the advantages and disadvantages of e-learning in the medical education system. The advantages are discussed here first, followed by the challenges.

Attitudes to e-learning and adaptability was one of the main advantages. The COVID-19 pandemic has been a major motive for developing their e-learning knowledge and skills. Given teachers’ obligations to deliver e-learning and provide the required training, relative success was obtained in improving skills and e-literacy for most people. Faculty members abandoned their previous attitudes toward education (i.e. their preference for verbal face-to-face instruction), which had caused reluctance to adopt new methods and technologies [29]. Among students also, the transition from classical to virtual instruction led them to improve their skills in working with different software, downloading electronic contents, and participating in e-learning. Some scholars have noted that students need not only traditional clinical education, but also need to stay up to date in recent technologies to support flexibility in a dynamic workplace [29]. Some believe that because of the conditions arising from the coronavirus pandemic, students, physicians, and teachers are constantly updating their skills to adapt to the changing health care environment and keep their digital literacy up to date [30].

The COVID-19 pandemic and the shift from traditional to virtual education have caused instructors and students to become quickly caught up in virtual education, with some learning quickly and others needing more help. The epidemic somehow forces people to adapt to existing and emerging policies, which leads to a change in their perception and ultimately leads to their greater adaptability.

At the beginning of lockdown, the unfamiliarity of some teachers with e-learning was associated with resistance to using it, but by providing appropriate infrastructure and improving teachers’ competencies, their participation in preparing and presenting virtual content increased. Moreover, as teachers became accustomed to e-learning and learned to match the contents to the curricula, initial resistance gradually decreased as e-learning became more widely accepted.

Negative attitudes and resistance have been shown to be obstacles to students’ involvement in e-learning [29]. Consequently, actions focused on removing these barriers can be vital for introducing successful e-learning [8] and eventually trickled into adaptability. Obliging university faculty members and students to move from traditional to virtual education can lead to better attitudes to e-learning and more adaptability, as long as support programs and appropriate opportunities and sufficient time are allowed for new skills to develop. In this connection, some research showed that engagement in e-learning activities can help faculty members obtain the required skills for teaching with new platforms [31], and thus lead to more flexibility and acceptance. According to some theories, people’s e-learning experiences are related with their behavioral intention to use e-learning [25]. Therefore, it is not surprising that teachers’ and students’ good experiences with e-learning can lead to more positive attitudes and acceptance of these new methods and technologies.

Participants said that while extensive closures of university facilities interrupted educational activities albeit only in the short term, simultaneous online and offline learning helped students feel less separated from the educational environment, and increased their academic engagement. In this connection, some studies have shown that e-learning, by enhancing students’ engagement [32] and self-directed learning opportunities [33], may increase their learning. According to Liaw et al., students’ engagement may be increased in the e-learning environment, and their problem-solving and critical thinking skills may consequently also improve [32]. These findings mean that through e-learning, students learn better by perceiving more engagement and feeling more connected to the learning environment.

Documenting and monitoring education was another major advantage. According to participants, although e-learning was implemented compulsorily, it provided an opportunity to involve both teachers and students in e-learning. It also resulted in closer monitoring of faculty members’ teaching activities and facilitated their evaluation.

Another advantage was the take control of own learning. Participants said that e-learning enabled them to save their lessons, including films, voiced slides and content delivery, so that they could review them as many times as they wished. The gain in taking control of own learning was likely a consequence of the longer-term availability of recorded classes [34]. Faculty members noted that preparing their classes for e-learning gave them time to revise the content and present it more coherently.

A study by Dyrbye et al. found that students preferred e-learning formats because of their flexibility and because they facilitated learning by providing opportunities for reflection on the course material [35]. A useful learning system can help students to manage and control their learning processes. For example, students can download and study learning materials in their own time and at their own pace. Even if students miss face-to-face classes, they can catch up on the course content online. Some studies found that when students were able to take more individual control over their learning, they achieved better learning results. This outcome suggests that an e-learning system may help students by increasing their control over their learning processes [36, 37].

The final advantage was perceived usefulness. According to the participants, the experience of e-learning changed their attitude towards the usefulness of this approach, and they perceived its usefulness more clearly than in the past. Perceived usefulness refers to the belief that e-learning systems increase a person’s job performance [38]. E-learning systems provide many features which may strengthen students’ learning or facilitate instruction. If students believe that e-learning formats enhance their learning, they are more likely to use e-learning [39, 40]. Therefore, a point worth highlighting is that the experience raised students’ and faculty members’ awareness of the benefits of e-learning and increased their positive attitude towards this approach. In this connection, studies on technology acceptance showed that perceived usefulness directly affected the use of educational technologies [20, 39, 41].

As noted above, our interviewees also noted that the COVID-19 pandemic had created some challenges to medical education. According to the qualitative analysis, these challenges were categorized into four subcategories.

Based on the experiences of most participants, students’ unfamiliarity with e-learning and their presence in virtual classes caused them to question how they should behave in this environment. Some individuals had experiences with inappropriate and unprofessional behaviors in the e-learning space. Issues with virtual learning etiquette may have been a consequence students’ unfamiliarity with novel e-learning approaches.

One of the biggest concerns of participants was to create effective interactions for maximum learning. Interviewees said that they did not have enough time for interaction and participation. In some courses, especially clinical and bedside education ones, a focus solely on e-learning was not considered effective or appropriate. Some studies have shown that e-learning can pose challenges to face-to-face interaction and participation. The lack of face-to-face interaction can be a barrier to building relationships, which can in turn lead to negative impacts on learning, performing educational tasks, and satisfaction [25, 35]. A study by Dyrbye et al. [35] showed that students needed immediate, real-time feedback from faculty members to confirm their perceptions and provide feedback on their efforts.

Another challenge was time limitations. According to faculty interviewees, providing daily up-to-date content takes longer than in-person education. Moreover, to deliver courses electronically, faculty members needed to spend more time on students’ learning, which created additional time pressures. Earlier studies have shown that medical faculty members face pressures to find enough time to manage teaching and research, and to achieve work–life balance [42]. Moreover, time allocations to learning, development, and using e-learning can further increase their time management challenges [29]. So, it’s not easy to save and invest time because computer-based tools require a lot of time [31]. For example; Dyrbye et al. [35] found that the faculty members had limited time to learn new technologies, and that time limitations led to concerns about educational and organizational aspects of e-learning. Some researchers have suggested that universities should provide specific times for faculty members to develop their skills and learn new technologies. Moreover, time shortages appear to be related with a lack of motivation to engage in online or electronic learning [43], and can become a barrier to using online teaching tools, especially if faculty members sometimes have to spend part of their allotted teaching time on such programs. Therefore, to ensure the effective use of these teaching tools, it is important to provide faculty members with opportunities to become familiar with and engage with these tools [29].

Infrastructural deficiencies and problems were other challenges mentioned by the participants. They reported unsatisfactory experiences with internet speed, uploading information on e-learning systems, downloading the contents, and weak support. As noted in the literature, weak infrastructure can prevent instructors from developing e-learning [35]. In most cases, infrastructural shortages and weaknesses may be a barrier to medical education [35, 44], especially in developing countries. For example, various studies have shown that slow internet speed and limited access, poor web quality, inadequate computer hardware, and lack of access to physical infrastructure may pose barriers [35, 44–46]. In this connection, Lakbala’s study in the Iran medical science context showed that limited access to computers, lack of support, limited support infrastructure, and high costs were among the main infrastructural barriers to e-learning [47]. Thus, infrastructural problems and deficits appear to negatively affect attitudes towards using e-learning. As indicated in numerous studies, system quality can directly and significantly affect the tendency to use e-learning [23–25].

Finally, the third main category extracted from the qualitative analysis was e-learning evaluation. Participants believed that virtual evaluation was more suitable for diagnosis and formative evaluation, which was done permanently. Yet in the case of summative evaluation, different views were expressed regarding the effectiveness of e-learning. According to the interviewees, given the possibility of group consultation on the answers (as a type of cheating), a mechanism should be provided for virtual individual evaluation. One suggested methods was holding open-book tests.

### Research limitations

There are several limitations in this study. First, the generalizability of the findings may be limited to our research sample because the study was conducted at a single university. Second, the researchers are engaged in e-learning programs and may have been biased in favor of specific responses by interviewees based on their experiences, or may have sought confirmation of their own attitudes and beliefs.

## Conclusion

Given the effect of the COVID-19 pandemic on all aspects of human life, especially the higher education system and medical education, medical schools have necessarily moved towards e-learning to compensate for the interruption in traditional methods of education by the replacement of traditional classes with e-learning. These rapid, extensive changes in teaching and learning styles have had consequences for medical schools.

Consistent with some previous studies, this study found evidence that e-learning can offer opportunities and achievements in medical education, such as improvements in attitudes to e-learning and adaptability, preventing students’ separation from the educational environment, take control of own learning, and documentation and monitoring education. These changes were accompanied by challenges such as inadequate interactions, time limitations, infrastructural deficits, and other issues.

### Study suggestions

In order to make e-learning more effective during the COVID-19 pandemic, it is suggested that colleges, while laying the groundwork for the effective use of e-learning by students and faculty, determine the rules and requirements for holding virtual classes which provide the necessary standards for effective and quality assurance of virtual classes, and inform faculty and students consequently.

Also, holding special training courses to empower professors and students in virtual education is of utmost importance. In this regard, it seems necessary to consider individuals or centers for technical support and troubleshooting and additional training for all involved in the process of e-learning. Another important suggestion is for departments and professors to try to take advantage of the opportunity to collaborate and network with their counterparts at other universities in their virtual education.

Due to the limitations of interactions in e-learning, it is suggested that professors use a variety of interactive software to actualize more student participation. Also, creating web groups to discuss educational topics can provide more opportunities for students to participate and engage. Creating and developing the necessary infrastructure for quality education, including providing and supporting desirable educational software, increasing and improving the speed of the Internet, granting free Internet packages to professors and students can improve virtual education. Finally, it is suggested that professors conduct formative assessments during semesters rather than judging student performance solely on the basis of a final assessment. Also, equipping colleges with up-to-date software and facilities for virtual evaluation of students can alleviate concerns in this regard.

## Data Availability

The datasets used during this study are available from the corresponding author on reasonable request.
